# Editorial: Advancements in Sepsis Diagnosis Utilizing Next-Generation Sequencing Approaches for Personalized Medicine

**DOI:** 10.3389/fcimb.2026.1839007

**Published:** 2026-04-28

**Authors:** Jitendra Kumar Tripathi, Kuldeep Gupta, Anupam Jyoti

**Affiliations:** 1 Translational Pulmonary and Immunology Research Center, Long Beach, CA, United States; 2 College of Medicine Phoenix, University of Arizona, Phoenix, AZ, United States; 3 Department of Life Science, Parul Institute of Applied Sciences, Faculty of Applied Sciences, Parul University, Vadodara, Gujarat, India

**Keywords:** diagnosis, machine learning, next-generation sequencing, omics, personalized medicine, sepsis

Sepsis is one of the leading causes of morbidity and mortality worldwide (Grey et al.) (Collaborators, 2025). It is a complex clinical syndrome resulting from host immune dysregulation in response to infection. It is characterized by multiple manifestations, resulting in dysfunction or failure of one or more organs and, in some cases, death. Early diagnosis is key to initiating timely intervention, as each hour of delayed treatment increases patient mortality by 7% (Kumar et al.) (Kumar et al., 2006). However, the diagnosis of sepsis is complicated by its heterogeneity, nonspecific clinical symptomatology, high-false negative rate, and overlapping clinical features with sterile inflammation. Additionally, the conventional methods have limited sensitivity, and prolonged turnaround time further delays the initiation of appropriate treatment. The advent of next-generation sequencing (NGS) technologies offers a promising solution to these diagnostic challenges, as it can analyze genetic material quickly and thoroughly.

The present Research Topic aims to explore the recent developments in NGS and related advanced molecular techniques in the diagnosis of sepsis. By fostering an interdisciplinary approach, the goal is to integrate studies focusing on the development of diagnostic biomarkers, molecular signatures, and OMICS and machine learning approaches as well as ensemble classifiers for diagnosis, severity, and predicting mortality with an aim of individualized treatment for sepsis patients. Additionally, the goal is to learn how OMICS-based biomarkers and machine learning utilizing large datasets help to develop new diagnostic models by fostering diagnostics and personalized medicine.

Metagenomic NGS (mNGS) is preferred over body fluid culture as it provides information at the species level without the need to culture. In a meta-analysis, Zhang et al. demonstrated that mNGS exhibits better sensitivity and specificity in detecting spinal infections as compared with routine microbial culture, highlighting it as a complementary diagnostic tool. Furthermore, mNGS outperforms the conventional methods in diagnosing central nervous system infections by offering rapid and accurate pathogen detection, better sensitivity, and less turnaround time (Cao et al.). Zhang et al. reported the improved performance of plasma cell-free DNA in pathogen detection in pediatric patients with hematologic diseases, enabling clinicians to initiate appropriate antimicrobial therapy and improve patient outcomes. Moreover, Pinzauti et al. utilized PISTE, an NGS-based technique for the detection and prediction of antimicrobial resistance in bloodstream pathogens in sepsis. It reduced the turnaround time and provided a reliable diagnostic tool for bloodstream infections.

Targeted NGS (tNGS) is an advanced technique utilizing targeted amplification and sequencing that can detect DNA and RNA pathogens simultaneously. In a study conducted by Ding et al., a comparison was made between tNGS and mNGS in detecting lower respiratory tract infections. tNGS showed comparable diagnostic efficacy to mNGS with a specific advantage in detecting fungi. Jin et al. utilized tNGS in detecting upper respiratory tract infections and found tNGS superior in terms of broader pathogen detection as compared with FilmArray.

Molecular diagnostic techniques are instrumental in the detection of pathogens associated with sepsis/other related diseases. Gao et al. performed pathogen detection in bronchiectasis utilizing tNGS, mNGS, and multiplex fluorescence quantitative PCR (qPCR) and compared it with conventional microbiological testing. Molecular diagnostics showed better efficacy for the detection of bronchiectasis pathogens as compared with routine culture methods. These techniques identify pathogen-specific clinical phenotypes and guide clinicians to administer appropriate anti-microbial therapies. Another breakthrough work was reported by Guo et al., who utilized sequencing technologies to characterize the intratumoral microbiota of cervical cancer. They observed an abundance of *Pseudomonas* in cervical cancer tissue, which induces the upregulation of fibrinogen beta chain gene and thereby promotes cell proliferation and migration.

Identification of gene signatures and prognostic classifiers utilizing transcriptomics and machine learning are instrumental in understanding and deciphering host response heterogeneity. Liu et al. identified three leptin-associated sepsis subtypes using non-negative matrix factorization. TFRC and PILRA were identified as potential biomarkers and further validated using qPCR and Western blotting. Shi et al. utilized an integrated machine learning approach by constructing an XGBoost algorithm for predicting the detection of *Staphylococcus aureus* bloodstream infections involving five genes (*DRAM1*, *UPP1*, *IL18RAP*, *CLEC4A*, and *PGLYRP1*).

Bulk transcriptomic and single cell data analysis can be used to decipher immune dysregulation pathways. Wang et al. identified key necroptosis genes using an integrated bioinformatics approach and concluded their role in the pathophysiology of sepsis and related immune responses. In another study conducted by Lu et al., two ferroptosis-associated biomarkers named DPP4 and TXN were identified using machine learning models and validated using RT-qPCR in sepsis samples. Jiang et al. utilized an omics followed by machine learning approach to identify *CX3CR1*, *PID1*, and *PTGDS*, which were then validated by RT-qPCR as key molecular signatures for the diagnosis of sepsis and acute respiratory distress syndrome. Furthermore, in a retrospective study, CRISP3 was found to be upregulated in adult sepsis and so bears potential as a latent biomarker (Zhang et al.).

Biomarkers help in early diagnosis, disease severity, and prediction of mortality in critically ill patients with sepsis. Lee et al. compared the levels of two plasma proteins, EphA2 and Del-1, in sepsis patients. EphA2 levels increased and correlated with sepsis severity, suggesting a better biomarker as compared with Del-1. Zhou et al. identified mitochondrial-encoded NADH dehydrogenase 6 (MT-ND6) and Annexin A1 as mortality-associated biomarkers and mixed inflammation phenotypes. Pu et al. reported that the prediction of pediatric sepsis improves when nucleated red blood cell counts are combined with C-reactive protein and pro-calcitonin.

In a retrospective analysis consisting of 490 patients over 10 years, a nomogram with four clinical features, namely white blood cell count, international normalized ratio, gas formation, and SOFA score, was developed to predict sepsis risk in pyogenic liver abscess (Zhang et al.). Gao et al. concluded the specific combinations of pathogens that influence survival in post-sepsis persistent respiratory dysfunction.

OMICS-based biomarkers have substantially deepened our understanding of sepsis by enabling comprehensive, system-level insights into host responses. Despite their promise, their integration into routine clinical practice remains limited compared with established molecular techniques, such as PCR and immunoassays. Key challenges include longer turnaround times, high costs, and the need for specialized infrastructure and bioinformatics expertise, all of which limit their utility in acute, time-sensitive settings. In addition, the complex and high-dimensional nature of OMICS data poses significant difficulties in interpretation, standardization, and reproducibility across studies. Patient heterogeneity further complicates the identification of robust and universally applicable biomarkers. In contrast, conventional molecular diagnostics offer faster, more accessible, and clinically validated solutions with clearer interpretability, making them more suitable for immediate clinical decision-making. Thus, while OMICS technologies hold significant potential for advancing precision medicine in sepsis, further efforts are required to improve their scalability, standardization, and clinical applicability.

Overall, these nineteen submissions in this Research Topic highlight a paradigm shift from conventional techniques toward modern techniques, including omics-based approaches. These studies also highlight the importance of machine learning, ensemble classifiers, and other computational approaches for the diagnosis of sepsis, its risk stratification, and predicting mortality with an aim of achieving personalized medicine or precision therapy for sepsis.
